# A systematic review of the factors associated with malaria infection among forest rangers

**DOI:** 10.1371/journal.pone.0303406

**Published:** 2024-05-15

**Authors:** Rahmat Dapari, Muhamad Zazali Fikri Mohd Yusop, Dharsshini Chinnasamy, Nurul Izati Zakaria, Siti Munisah Mohd Shoaib, Mohd Erfan Edros

**Affiliations:** Department of Community Health, Faculty of Medicine and Health Sciences, Universiti Putra Malaysia Serdang, Selangor, Malaysia; Fundação Oswaldo Cruz Centro de Pesquisas René Rachou: Fundacao Oswaldo Cruz Instituto Rene Rachou, BRAZIL

## Abstract

**Introduction:**

Malaria is a vector-borne disease that initially manifests as fever, headache, and chills. The illness could progress to more severe conditions, including lethargy, impaired consciousness, convulsions, shortness of breath, blood in urine, jaundice, and haemorrhage if left untreated. The risk of contracting malaria is considerably heightened in specific occupational settings, particularly among forest rangers, following frequent exposure to natural habitats. Consequently, advancing the understanding of malaria and emphasising how specific occupational environments (including those of forest rangers) contribute to disease risk and management is imperative.

**Objective:**

The present study aims to determine the factors associated with malaria infection among forest rangers by systematically reviewing electronic articles from three databases (EBSCOhost, ScienceDirect, and ResearchGate).

**Methods:**

The current review was prepared based on the updated preferred reporting items for systematic reviews and meta-analyses (PRISMA) guidelines. First, three independent reviewers screened the titles and abstracts of the data collected. The information was then stored in Endnote20 based on the inclusion and exclusion criteria. The articles were critically appraised with the mixed methods appraisal tool (MMAT) to assess their quality.

**Result:**

A total of 103, 31, and 51 articles from EBSCOhost, ScienceDirect, and ResearchGate, respectively, were selected, resulting in 185 unique hits. Nevertheless, only 63 full-text publications were assessed following a rigorous selection screening, from which only five were included in the final review. The studies revealed that several factors contribute to malaria infection among forest rangers. The parameters were classified into sociodemographic, individual, and living condition-related.

**Conclusion:**

A better understanding of malaria progresses and identifying its potential risk factors is essential to impact worker well-being. The findings might be utilised to improve malaria infection prevention programme implementations, hence maximising their success. Pre-employment and regular health screenings could also aid in evaluating and identifying potential risks for malaria infection among forest rangers.

## Introduction

The World Health Organization (WHO) reported that in 2022, global malaria cases surged to approximately 249 million across 85 countries and regions where malaria is endemic, including French Guiana. This marks a rise of 5 million cases from 2021. Pakistan, Ethiopia, Nigeria, Uganda, and Papua New Guinea were significant contributors to this increase, with Pakistan seeing a rise of 2.1 million cases, Ethiopia and Nigeria each experiencing a surge of 1.3 million cases, and Uganda and Papua New Guinea witnessing increases of 597,000 and 423,000 cases respectively [1]. Despite this rise, incidence rates have remained constant over the past three years, with 58 cases reported per 1000 population at risk in 2022 [1]. Malaria, a vector-borne disease, is transmitted by infected Anopheles mosquitoes. Nevertheless, transmissions through blood transfusion and contaminated needles have also been reported. *P*. *falciparum*, *P*. *vivax*, *P*. *malariae*, *P*. *ovale*, and *P*. *knowlesi* are the five parasites responsible for malaria infections. Nonetheless, only *P*. *falciparum* and *P*. *vivax* pose a serious threat. The malaria incubation period ranges from 10 to 15 days. The early symptoms of the disease are fever, headache, and chills, which could worsen without early treatment or if immunocompromised individuals are infected. The symptoms could evolve into extreme lethargy, impaired consciousness, convulsions, dyspnoea, haematuria, jaundice, and haemorrhagia [2].

Malaria is prevented by avoiding mosquito bites by employing vector control [insecticide-treated nets (ITNs) and indoor residual spraying (IRS)], chemoprophylaxis, and vaccines. Available vaccines include RTS, S/AS01, initiated in October 2021, and R21/Matrix-M, recommended in October 2023. The RTS, S/AS01 vaccine has significantly reduced malaria transmission since its initiation [3].

Malaria remains a formidable public health challenge, particularly in regions where forested environments harbour the vectors responsible for transmission [4]. Among those at heightened risk are forest rangers, whose duties often entail prolonged exposure to these vector habitats [5]. Although there have been concerted efforts to combat the disease, malaria continues to exact a heavy toll on the health and productivity of populations in forested areas, impeding conservation efforts and jeopardizing their well-being [6]. Despite the monumental strides made in malaria control efforts globally, including extensive distribution of insecticide-treated bed nets, indoor residual spraying campaigns, and widespread availability of antimalarial drugs, the complex ecological and socio-economic factors influencing malaria transmission persistently thwart eradication efforts [7, 8]. Particularly for populations like forest rangers who often inhabit remote and densely forested areas, access to preventative measures and healthcare services remains limited, rendering conventional control strategies insufficient to curb malaria incidence effectively [9]. Despite the extensive and comprehensive scope of malaria programs worldwide, the daunting challenge of eliminating malaria, especially among vulnerable groups like forest rangers, remains an elusive goal requiring innovative and targeted interventions.

Previous studies have confirmed that various factors, such as age, gender, marital status, family size, the use of Long-Lasting Insecticidal Nets (LLINs), Indoor Residual Spraying (IRS), proximity to mosquito breeding sites, and the presence of less robust and porous walls, play significant roles in determining infection rates [10]. Additionally, temperature, precipitation, relative humidity, wind intensity, and direction are identified as the most influential climatic factors affecting the growth and proliferation of Anopheles mosquitoes, Plasmodium parasites, and the burden of malaria [11]. Compounding these challenges, socioeconomic status, including lower income, house type, and distance to health sub-centers, has been found to be positively associated with malaria occurrence [12]. However, despite this wealth of information, the unique factors contributing to malaria among forest rangers remain scientifically uncertain.

The objective of this systematic review is to thoroughly investigate the factors linked to malaria infection among forest rangers. By synthesizing existing evidence and identifying key determinants of malaria transmission within this population, the study aims to elucidate the complex interplay between sociodemographic, individual, and living condition-related that contribute to the heightened malaria risk faced by forest rangers.

## Methodology

### Materials and methods

The present study followed the updated guidelines of preferred reporting items for systematic reviews and meta-analyses (PRISMA). This study aimed to identify the factors associated with malaria infection among forest rangers. The population, exposure, and outcome (PEO) components of the current study were established as follows.

- Population: Forest rangers
- Exposure: Factors associated with malaria infection
- Outcome: Malaria infection

### Search strategy

The literature search in this study was conducted from 15 November 2023 to 15 December 2023. The EBSCOhost, ScienceDirect, and ResearchGate databases were employed. This study also used “associated factor*” OR factor* AND “malaria infection*” OR malaria AND “forest ranger*” OR “forest goer*” OR “park ranger*” OR “forest worker*” as keywords during the search. All the retrieved articles were imported into the EndNoteX7 library before performing library de-duplication.

### Eligibility criteria

The present study only reviewed publications in English from 2017 and original articles, including cohort, case–control, and cross-sectional studies, investigating the associated factors for malaria infection. Articles that included forest rangers as the study population and malaria infection as an outcome were considered. Conversely, non-original articles involving conference proceedings, perspectives, commentary, opinions, reports, systematic reviews, and meta-analyses were excluded. The current eligibility criteria restrict publications from 2017 to the time of review (2023), encompassing a span of five years to prioritize recent data. This approach aims to ensure the inclusion of the latest advancements and findings in the field, thereby incorporating up-to-date evidence. Additionally, narrowing the search to the past five years streamlines the review process and maintains relevance to current malaria transmission patterns.

### Study selection

The present study involved three independent reviewers to screen the titles and abstracts of the retrieved materials against the inclusion and exclusion criteria. Subsequently, potential full texts of articles identified during the primary screening were reviewed independently by five independent reviewers following the inclusion and exclusion criteria. Any disagreements during the phase were discussed as a group among the five reviewers.

### Critical appraisal and data extraction

The mixed method appraisal tool (MMAT) was employed during quality appraisal. During the assessment, the quality of each article was evaluated based on methodological criteria, including five core quality parameters [13]. In this study, two reviewers extracted the data, which were then assessed independently by two other reviewers. Finally, eligible articles were analysed with the content analysis method without employing any statistical tests.

## Results

**Fig 1:**
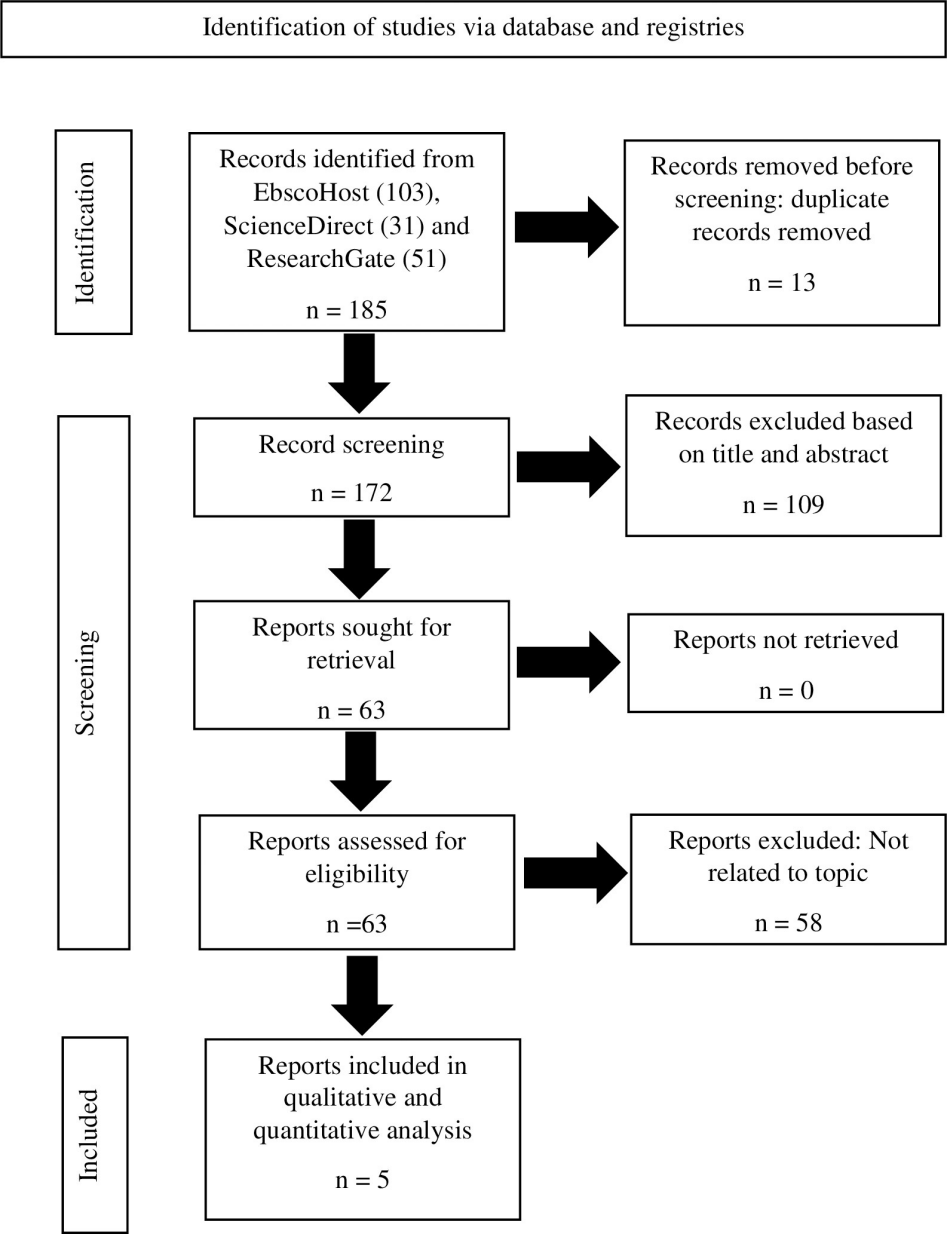
PRISMA flow diagram for the systematic review.

The search in this study yielded 103 articles from EBSCOhost, 31 from ScienceDirect, and 51 from ResearchGate, totalling 185 unique hits (Fig 1). PRISMA 2020 flow diagram for new systematic reviews was used for this review [14]. Subsequently, these hits underwent a stringent selection screening process, where each article was meticulously evaluated for its relevance to selection criteria. Following this thorough assessment, only 63 full-text articles were identified as meeting the criteria for further screening process. Following this thorough assessment, 58 articles were removed as they did not meet the inclusion criteria, either due to the study population, exposure or outcome not being related to the focus of this study review. This meticulous approach ensured that only the most pertinent and high-quality literature was included in our review, thereby enhancing the robustness and validity of our findings. Table 1 lists the details of the selected publication, while Table 2 summarises the findings reported by the five studies reviewed. The articles in this study were published between 2019 and 2023. Two articles were cross-sectional studies, two was a qualitative study, and another was a case-control study. Any duplications were removed with automated removal in EBSCOhost.

**Table 1:** The selected study location and study design details.

Authors	Study location	Study design
Ekawati et al. (2020) [15]	Aceh, Indonesia	Qualitative
Doum et al. (2023) [16]	Mondulkiri, Cambodia	Cross-sectional
Istiana et al. (2021) [17]	South Kalimantan, Indonesia	Cross-sectional
Canavati et al. (2019) [18]	Phu Yen Province, Vietnam	Case-control
Jongdeepaisal et al. (2022) [9]	Lao PDR	Qualitative

**Table 2:** The selected publications summary.

Author (Year)	Title	Study design	Sample size	Outcome/Method of assessment	Factors
Ekawati et al. (2020) [15]	Defining malaria risks among forest workers in Aceh, Indonesia: A formative assessment	Qualitative study	231	Index cases, co-workers, community health workers, residents of forest fringe villages, non-forest fringe village residents, *tokes*, community leaders, government authorities, and health facility staff were involved.	• Extended stays in forests. • Lack of prevention practice. • Misconceptions and beliefs regarding malaria. • Delayed treatment-seeking behaviour. • Risk behaviours include • not using mosquito nets or repellent at night, • working and sleeping outdoors at night, and • only lighting a fire to ward off mosquitoes when sleeping. • Practical challenges when implementing preventive measures.
				The 72 IDIs and 20 FGs were analysed thematically following a grounded theory approach to identify key themes and patterns related to malaria risks and treatment-seeking behaviours among forest workers. The study also involved the development of a draft codebook for qualitative analysis and the utilisation of qualitative analysis software for data coding.	
Doum et al. (2023) [16]	An active and targeted survey reveals asymptomatic malaria infections among high-risk populations in Mondulkiri, Cambodia	Cross-sectional	1301	The participants were recruited from 21 villages and 15 ranger stations in the Mondulkiri province.	Gender (socio-demographic) factor. Females had less odds contracting malaria than males (aOR = 0.61; 95% CI: 0.38–0.97, p = 0.04)
				Individuals with significant malaria infection risk were targeted for blood sample collection. The participants include	
				1. forest rangers and patrol teams spending at least 21 days or a month in forested areas,	
				2. forest dwellers, including those living and working in forests or within 1 km of forest edges, and	
				3. forest goers living over 1 km from forest edges but visit forested areas at least a day or a week.	
				The outcome measure was the malaria infection prevalence (by parasite species) per target population and was determined using the number of positive individuals divided by the total sample size.	
Istiana et al. (2021) [17]	Malaria at Forest Areas in South Kalimantan, Indonesia: Risk Factors and Strategies for Elimination	Cross-sectional	107	A standardised questionnaire was employed to collect patient age, gender, village of residence, education, occupation, ethnicity, and house condition data, including roof condition, walls, house ceiling, mosquito wire screen utilisations, and animal cage possession.	• Age: Participants under 25 years old recorded a higher prevalence of malaria (OR = 2.526; 95% CI: 0.804–7939) • Occupation: Forest workers documented an increased risk of malaria (95% CI: 0.572–47.169) • Housing conditions: • Living in houses with an open wall: (aOR = 4.677; 95% CI: 0.533–41.079) • Living in houses with unstandardised roofs: (aOR = 3.025; 95% CI: 0.761–12.024) • Animal cages around the house: (OR = 6.292)
				Blood samples were obtained from the participants for malaria microscopy and rapid diagnostic evaluations.	
Canavati et al. (2019) [18]	Risk factor assessment for clinical malaria among forest-goers in a pre-elimination setting in Phu Yen Province, Vietnam	Case-control study	81 cases and 94 controls, approximately 1:1 case to control ratio	Participants were interviewed face-to-face using a standard questionnaire to identify malaria risk factors.	Utilisation of treated nets (OR = 0.31; 95% CI: 0.12–0.7) Sleeping in huts without walls (or was not computed due to the low number of observations, n = 4) Collecting water after dark (aOR = 1.99; 95% CI: 1.02–3.90) Bathing in streams after dark (aOR = 2.44; 95% CI: 1.02–5.88) Working after dark (aOR = 2.93; 95% CI: 1.35–6.34)
Jongdeepaisal et al. (2022) [9]	Forest malaria and prospects for anti-malarial hemoprophylaxis among forest goers: Findings from a qualitative study in Lao PDR	Qualitative study	31	In-depth interviews were conducted with 16 forest goers and 15 stakeholders in Savannakhet province, Lao PDR	• Overnight in farm huts • Working and sleeping at night in forests • Spending time outdoors at home • Staying in houses close to mosquito breeding pools • Insufficient employment of insecticide-treated nets (ITNs)

### Sociodemographic parameters

A cross-sectional study in Cambodia revealed that females have a lower risk of contracting malaria than males (aOR = 0.61; 95% CI: 0.38–0.97, p = 0.04) [16]. Nonetheless, a study conducted in Kalimantan indicated no significant links between gender and malaria infection [17]. The report also revealed that individuals under 25 years old were more likely to be infected with malaria than their older counterparts (aOR = 2.526; 95% CI: 0.804–7939) [17]. Another study documented no remarkable association between age and malaria infection [16]. Ethnicity was also not a considerable factor in contracting malaria [16, 17].

Forest workers had higher risks of malaria infection than non-forest workers (95% CI: 0.57–47.17) [17]. A report noted that the type of work in the forest was not significantly associated with malaria infection [18]. A qualitative study in Lao PDR reported that the respondents were commonly involved in forest activities, such as collecting food and wild products, caring for cattle, and hunting. Some participants also reported spending a few days to a week in forests once or twice a month [9]. Nonetheless, among the 1301 respondents in the study, the frequency of travelling outside the village and working in the forest recorded no significant association with malaria infection [16].

### Individual factors

No considerable link was recorded between activities after dark and malaria infection among forest-goers in Vietnam [18]. Nevertheless, the study found that conducting tasks, such as collecting water (aOR = 1.99; 95% CI: 1.02–3.90), bathing in streams (aOR = 2.4; 95% CI: 1.02–5.88), and working after dark (aOR = 2.93; 95% CI: 1.35–6.34), increased chances of malaria infection [18].

Sleeping habits were significantly associated with malaria infection. A qualitative study in Aceh revealed that respondents perceived sleeping without bed nets or repellent at night as contributing to malaria [15]. Diminished malaria risks were notably associated with utilising treated nets (aOR = 0.31; 95% CI: 0.12–0.80). Nonetheless, sleeping with untreated or without bed nets was not significantly associated with malaria infection [18]. The respondents in another study recorded employing bed nets more frequently at home than in farm huts. The participants also indicated awareness of the importance of employing mosquito coils and repellent but could not afford the devices [9].

Most respondents in the articles reviewed in this study reported preferring to ignore symptoms or would self-treat with medications purchased from local pharmacies as a precaution when venturing into forests. The participants were also inclined to go to traditional healers or private clinics if the symptoms persisted after self-treatment. Reportedly, the respondents found that obtaining treatment in primary health centres is complicated. Moreover, the centres are commonly out of anti-malarial drugs [15]. Meanwhile, a study revealed that the respondents have never taken malaria prevention medication but would do so if it was free [9].

### Living-conditions linked components

Some living conditions could be linked to malaria infection. Studies conducted in Vietnam and Kalimantan indicated that sleeping in housing without walls was notably correlated to malaria infection (aOR = 4.677; 95% CI: 0.53–41.08) [17, 18]. Living in plastic-roofed houses considerably increased the risk of malaria infection (aOR = 3.03; 95% CI: 0.76–12.02) [17]. On the other hand, other living conditions, including house ceilings, mosquito wire screens, and water bodies, were not significantly linked to contracting malaria [17].

### Bias risks

The present study evaluated all five articles selected with the mixed methods appraisal tool (MMAT). The methodology quality of the publications was appraised based on five criteria [13]. Table 3 details the MMAT assessment of the reports reviewed.

**Table 3:** The MMAT assessment details.

Author	Type of study	Are the participants representative of the target population?	Are the outcome and intervention (or exposure) measurements appropriate?	Is the outcome data complete?	Are the confounders accounted for in the design and analysis?	During the study period, is the intervention administered (or exposure occurred) as intended?
Ekawati et al. (2020) [15]	Qualitative non-randomised	Yes	Yes	No	No	No
Doum et al. (2023) [16]	Quantitative non-randomised	No	Yes	No	Yes	No
Istiana et al. (2021) [17]	Quantitative randomised	No	Yes	No	Yes	No
Canavati et al. (2019) [18]	Quantitative non-randomised	Yes	Yes	No	Yes	No
Jongdeepaisal et al. (2022) [9]	Quantitative non-randomised	No	Yes	No	No	No

## Discussion

According to the articles reviewed in this study, several factors contribute to malaria infection among forest rangers. The parameters could be classified into sociodemographic, individual, and living-condition-related factors.

### Sociodemographic parameters

The publications reviewed in the current study indicated a significant association between several sociodemographic factors and malaria infection. For instance, a study noted that females have a lower risk of contracting malaria than males [16] which was similar to the findings of a study performed in northern Namibia suggested that the role of men as breadwinners might have exposed them to increased malaria infection risks based on their occupation types [19, 20]. Nonetheless, the article was excluded from the final review and Table 2 as it did not satisfy the inclusion and exclusion criteria outlined in this study. Nevertheless, an article revealed no significant association between gender and malaria infection [17]. The contradictory results might be due to differences in the sample size and distribution of male and female respondents.

Age was not significantly associated with malaria infection [16]. Nonetheless, other study indicated that individuals aged under 25 had increased chances of being infected with malaria [17]. This finding was supported by a report that revealed children aged 0–4 years old had a lower malaria infection risk than the older age categories [19]. However, this study was removed in the final review and Table 2 for not meeting the inclusion and exclusion criteria of this study. The articles reviewed in the current study also found no remarkable links between ethnicity and malaria infection [16, 17, 20].

In a study, it was noted that forest workers were more likely be infected with malaria than non-forest workers [17]. Individuals working outdoors might have diminished employment of malaria prophylaxis and miss intervention campaigns regularly [19]. Forest workers are also more exposed to the illness due to the disruption of mosquito habitat following deforestation [20]. Furthermore, the respondents collect food and wild products, care for their cattle, and hunt, requiring them to spend few days to a week once or twice a month in the forest [9]. Nevertheless, other study did not find a significant association between the frequency of travelling outside the village and working in the forest with malaria infection [16].

### Individual factors

Despite having similar sociodemographic and living condition factors, individual components also influence the risks of malaria infection. Several examples of the individual factors were after-dark activities, sleeping habits, and health-seeking behaviours. Adult female Anopheles mosquitoes typically bite humans late in the evening or at night [21]. Consequently, individuals engaging in activities after dark are exposed to the insects and have an increased chance of being infected with malaria. The articles reviewed indicated that several activities were significantly related to malaria infection, including collecting water (aOR = 1.99; 95% CI: 1.02–3.90), bathing in streams (aOR = 2.4; 95% CI: 1.02–5.88), and performing tasks after dark (aOR = 2.93; 95% CI: 1.35–6.34) [18].

Malaria is inextricably tied to water as Anopheles mosquitoes utilise water bodies for breeding. The common feature of Anopheles mosquito habitats is stagnant or very slow water flow, including water leakage and fast-flowing margins [22]. Individuals engaging in activities near the water bodies are exposed to mosquitoes, increasing their likelihood of being bitten and infected with malaria. Parameters primarily related to activity patterns are vital for understanding the points of human exposure to malaria vectors. The factors could also assist local authorities, especially those of public health interest, in pursuing suitable health promotional campaigns tailored to local requirements.

A study hypothesised that several habits might predispose humans to mosquito bite, including sleeping without net (aOR = 0.31; 95% CI: 0.12–0.80) and insect repellent [15]. In another study, malaria infection risks were reduced in homes with adequate household net coverage and sleeping under a net (aOR = 0.63 95% CI 0.42–0.94 and aOR = 0.61 95% CI 0.42–0.87) [19]. The findings suggested that the effects of household-level protection persist after accounting for individual net utilisation. Conversely, a study in Saudi Arabia revealed that sleeping habits were not significantly associated with malaria infection [23].

The articles reviewed in the present study indicated that the health-seeking behaviour of individuals was considerably related to malaria infection. Among the behaviours highlighted in the publications were ignoring malaria symptoms, self-treating with medications purchased from local pharmacies, and seeking treatments from traditional healers or private clinics [15]. On the other hand, 35% of the respondents in a study in Ghana preferred self-prescribed antimalaria treatments [24]. Reports have documented widespread inappropriate malaria treatment-seeking behaviour. Nevertheless, improving accessibility to malaria treatment services and reducing wait time and treatment costs at health facilities could mitigate inappropriate health-seeking behaviours. The association between health-seeking behaviour and malaria infection emphasises the crucial role of proactive healthcare-seeking actions in preventing, diagnosing, and treating the illness. Furthermore, effective strategies in promoting positive health-seeking behaviours are vital to reduce the malaria burden malaria and improve the health outcomes of affected communities.

### Living-condition related components

Living conditions of forest rangers, including their houses and the environment around their living areas, have notable associations with malaria infection. Among the factors contributing to the link are living in homes or huts without walls or unstandardised roofs and having animal cages or close proximity to mosquito breeding pools.

Forest rangers living in houses or huts without walls or with unstandardised roofs have increased likelihood of contracting malaria [17, 18]. Plastic roofs also contributed to increased risks of malaria infection than their zinc counterparts. Plastic roofs are temporary protection that is prone to damage following weather changes, such as sun exposure and heavy rain, resulting in mosquito entry into the house [17]. Another study also demonstrated that traditionally constructed homes and sleeping in open structures enhanced the chances of malaria infection [19].

Significant correlation was identified between owning animal cages in houses and malaria infection. It was revealed that livestock attract mosquitoes, as evidenced by chicken coops and cattle sheds placed two to three metres from houses, thus contributing to the increased risks of contracting malaria [17]. Although some studies reported that rearing livestock could reduce Anopheles mosquito bites by providing zooprophilic protection, some reports reported otherwise [25, 26].

Despite the wealth of research on malaria transmission in endemic regions, there exists a notable gap in understanding the specific determinants of malaria infection among forest rangers. While individual studies have examined various factors in isolation, there is a lack of comprehensive synthesis and analysis to discern the collective impact of these factors within the unique context of forest ranger populations. This gap hampers the development of targeted interventions tailored to the specific needs of forest rangers, impeding efforts to mitigate malaria risk in this vulnerable demographic.

The findings of this systematic review hold significant implications for malaria control efforts targeting forest ranger populations. By elucidating the factors contributing to malaria transmission among forest rangers, our study seeks to inform the development of evidence-based interventions aimed at reducing malaria burden and improving the health outcomes of this vulnerable group. Furthermore, by identifying gaps in current knowledge, this review will guide future research endeavors and policy initiatives aimed at safeguarding the health and well-being of forest rangers in malaria-endemic regions.

## Recommendations

Comprehensive training programmes emphasising the importance of personal protective measures and early symptom recognition should be developed to address malaria infection among forest rangers. District-level health facilities and microscopic malaria diagnosis trained staff are also necessary. Moreover, designing interventions that address specific needs, including mobile malaria health workers providing diagnostics and treatment in remote areas. The strategies could provide forest goers access to timely and accurate diagnosis and treatment. Regular health screenings and essential protective gear provision, such as insecticide-treated nets and repellents are also crucial.

Environmental control strategies should be researched to minimise mosquito breeding near work and living areas. Moreover, investigating behavioural change campaigns efficacy and access to prophylaxis in high-risk zones is required. Improving living conditions to prevent mosquito entry and continuously monitoring malaria incidences among rangers are also vital. Collaborative efforts with health authorities and local community engagement could amplify the impact of the measures. The comprehensive approach and integration of practical interventions with ongoing research might offer a promising pathway for reducing malaria risks among forest rangers.

## Limitations

The present study revealed limitations, including publication bias due to the exclusion of grey literature. Although the search strategy in this review resulted in articles from several countries where English is not the primary language (such as France), only articles in English were selected. Consequently, the present study could have language bias. Despite the limitations, this study successfully synthesised research evidence on the factors associated with malaria infection among forest rangers. The current study potentially serves as a guide to improve malaria prevention strategies and programmes among forest rangers.

## Conclusion

Managing malaria infections is critical to better understand the development of the disease and its potential risk factors, which affects the forest rangers well-being. Knowledge of malaria infection determinants could also permit the implementation of effective preventative measures. Consequently, understanding the factors influencing malaria infection is critical. The findings might be utilised to improve malaria infection prevention programme implementations, hence maximising their success.

## Supporting information

S1 Checklist PRISMA 2020 checklist. (DOCX)

S1 File (PDF)
