# Supplementary material for: A systematic review of the factors associated with malaria infection among forest rangers
Source: PLoS One. 2024 May 15;19(5):e0303406. doi: 10.1371/journal.pone.0303406 (PMC11095669; doi:10.1371/journal.pone.0303406)
Supplement: S1 File — (PDF) [file pone.0303406.s002.pdf]

Dr. THAVAMARAN KANESAN
Chief Editor
Company: Proofreading by A UK PhD (ProofreadingByPhD.com)
Registration: NS0163595-K
Email: DrThava@proofreadingbyphd.com
Mobile: +60176712515

PROOFREADING BY A UK PHD

PROFESSIONAL PROOFREADING CERTIFICATE

This document certifies that the material detailed below underwent a thorough proofreading process, and was subsequently improved in terms of English language, grammar, punctuation spelling, overall styling and quality-of-expression.

Title :
A Systematic Review of the Factors Associated with Malaria Infection Among Forest Rangers

Authors :
Dharsshini Chinnasamy[1], Muhamad Zazali Fikri Bin Mohd Yusop[1], Nurul Izati Binti Zakaria[1], Siti Munisah binti Mohd Shoaib[1], Mohd Erfan Bin Edros[1], Rahmat Dapari[1]

Affiliations :
[1]Department of Community Health, Faculty of Medicine and Health Sciences, 43400 Universiti Putra Malaysia Serdang, Selangor, Malaysia

Date Issued: 14 Feb 2024, Wed

Yours truly,

T. Kanesan

DR. THAVAMARAN KANESAN
Chief Editor @ Proofreading By A UK PhD
(NS0163592-K)
51-1, First Floor, Block G, Biz Avenue 2 @ Neocyber
Lingkaran Cyber Point Barat, 63000 Cyberjaya, Selangor
H/P: +6017 671 2515
www.proofreadingbyphd.com

sfep
society for editors and proofreaders

HRDF
REGISTERED
TRAINING PROVIDER

- **60,800+** Documents Proofread/Edited/Structured/Paraphrased
- **8,400+** Articles Accepted in SSCI/SCI/HSCI/Scopus/ERA
- **6,100+** Theses Passed Viva
- **500+** Grant Documents Approved
- **650+** CV and Research Statements Completed

STANDARDS MALAYSIA ACCREDITED CERTIFICATION BODY
QS 22062015 CB 19

Platinum Shauffmanz Veritas PSV CERTIFIED ISO 9001
MAS 401122

Quality Management System

Remaining documents are all under review
